# Methods and protocol of a mixed method quasi-experiment to evaluate the effects of a structural economic and food security intervention on HIV vulnerability in rural Malawi: The SAGE4Health Study

**DOI:** 10.1186/2193-1801-3-296

**Published:** 2014-06-12

**Authors:** Lance S Weinhardt, Loren W Galvao, Thokozani Mwenyekonde, Katarina M Grande, Patricia Stevens, Alice F Yan, Lucy Mkandawire-Valhmu, Winford Masanjala, Jennifer Kibicho, Emmanuel Ngui, Lindsay Emer, Susan C Watkins

**Affiliations:** Joseph J. Zilber School of Public Health, University of Wisconsin Milwaukee, PO Box 413, Milwaukee, WI 53201 USA; Center for Global Health Equity, College of Nursing, University of Wisconsin Milwaukee, PO Box 413, Milwaukee, WI 53201-0413 USA; CARE-International Malawi, CARE House, Kenyatta Road Capital City, Lilongwe, Malawi; College of Nursing, University of Wisconsin Milwaukee, PO Box 413, Milwaukee, WI 53201-0413 USA; Department of Economics, University of Malawi, Chancellor College, P.O. Box 280, Zomba, Malawi; University of Pennsylvania, Philadelphia, PA USA; California Center for Population Research, University of California-Los Angeles, 4284 Public Affairs Bldg, PO Box 957236, Los Angeles, CA 90095-7236 USA

**Keywords:** Food security, Microfinance, Village savings and loans, HIV, Quasi-experimental design, Malawi

## Abstract

**Background:**

Poverty and lack of a predictable, stable source of food are two fundamental determinants of ill health, including HIV/AIDS. Conversely, episodes of poor health and death from HIV can disrupt the ability to maintain economic stability in affected households, especially those that rely on subsistence farming. However, little empirical research has examined if, and how, improvements in people’s economic status and food security translate into changes in HIV vulnerability.

**Methods:**

In this paper, we describe in detail the methods and protocol of an academic-NGO collaboration on a quasi-experimental, longitudinal study of the mechanisms and magnitude of the impact of a multilevel economic and food security program (Support to Able-Bodied Vulnerable Groups to Achieve Food Security; SAFE), as implemented by CARE. Primary outcomes include HIV vulnerability (i.e., HIV risk behaviors, HIV infection), economic status (i.e., income, household assets) and food security (including anthropometric measures). We recruited participants from two types of areas of rural central Malawi: traditional authorities (TA) selected by CARE to receive the SAFE program (intervention group) and TAs receiving other unrelated CARE programming (controls). In the intervention TAs, we recruited 598 program participants (398 women, 200 men) and interviewed them at baseline and 18- and 36-month follow-ups; we interviewed 301 control households. In addition, we conducted random surveys (n = 1002) in the intervention and control areas with a 36-month assessment interval, prior to and after implementation of SAFE. Thus, we are examining intervention outcomes both in direct SAFE program participants and their larger communities. We are using multilevel modeling to examine mediators and moderators of the effects of SAFE on HIV outcomes at the individual and community levels and determine the ways in which changes in HIV outcomes feed back into economic outcomes and food security at later interviews. Finally, we are conducting a qualitative end-of-program evaluation consisting of in-depth interviews with 90 SAFE participants.

**Discussion:**

In addition to examining pathways linking structural factors to HIV vulnerability, this research will yield important information for understanding the impact of a multilevel environmental/structural intervention on HIV, with the potential for other sustainable long-term public health benefits.

## Background

HIV/AIDS, poverty, and food insecurity contribute substantially to morbidity and mortality in sub-Saharan Africa (Masanjala 2007; Tsai et al. 2011). The Republic of Malawi, in southeastern Africa, bears one of the heaviest HIV disease burdens globally, ranking ninth worldwide for prevalence of HIV/AIDS among adults (ages 15–49) (Central Intelligence Agency CIA 2010). The 2010 Malawi Demographic and Health Survey (MDHS) (National Statistical Office NSO and ICF Macro 2011) reported an adult HIV prevalence rate of 11%, with a disproportionately higher rate for women than for men (13% versus 8%; (National Statistical Office NSO and ICF Macro 2011). Poverty is endemic in Malawi; more than half of its estimated 15 million people live on less than a dollar a day (Masanjala 2007; Integrated Household Survey IHS3 2010-2011). Food insecurity, defined as having uncertain or limited access to nutritionally adequate food, or being unable to procure food in socially acceptable ways (Anderson 1990; Olson 1999), is an aggravated problem in Malawi. The latest Integrated Household Survey (IHS3, 2010–2011) showed that half of Malawian households (49%) faced food shortages in the year preceding the survey. Taken together, Malawi seems to be caught in a vicious cycle wherein poverty and food insecurity increase people’s vulnerability to HIV infection, while HIV infection reduces peoples’ ability to earn money and produce food (Aarehag et al. 2006; Bryceson et al. 2004; de Waal and Whiteside 2003).

To better understand the context of HIV in Malawi, and to determine potential responses, it is important to consider HIV within an ecosocial framework (Krieger 1994; Loevinsohn and Gillespie 2003). Moving beyond the conventional focus on proximal factors contributing to HIV vulnerability, like individual risk behaviors, it is essential that interventions address poverty and food insecurity (Anema et al. 2009; Miller et al. 2011) as interrelated distal factors in the HIV pandemic, especially in countries like Malawi. Poverty has been consistently recognized as a risk factor for food insecurity (Maes et al. 2010; Nelson et al. 1998; Normén et al. 2005). Focusing on poverty, the Intervention with Microfinance for AIDS and Gender Equity (IMAGE) study, which combined a microfinance program with a gender and HIV training curriculum, was associated with decreased levels of self-reported intimate partner violence and unprotected sex (Kim et al. 2007; Pronyk et al. 2006).

Food insecurity, in turn, is associated with decreased adherence to HIV antiretroviral therapy (Weiser et al. 2009a, b), incomplete virologic suppression (Wang et al. 2011; Weiser et al. 2009a), declines in physical health status, (Weiser et al. 2009a, 2010), worse immunologic status (Kalichman et al. 2010; Weiser et al. 2009b), increased incidence of serious illness (Weiser et al. 2010) increased mortality (Anema et al. 2013) and decreased survival (Anema et al. 2013). In particular, a cross-sectional study in Brazil found that severe food insecurity with hunger is associated with reduced condom use and with increased occurrence of symptoms that may indicate sexually transmitted disease among sexually active women (Tsai et al. 2012). These findings indicate that HIV prevention strategies in food insecure areas should include interventions that target food insecurity.

Increasing critique has targeted the limitations of proximally focused HIV prevention interventions (Weinhardt et al. 1999; Johnson et al. 2003) and pointed out the need for development and assessment of complex, multilayered structural interventions (Gupta et al. 2008) that address root causes and causal pathways linking social, economic, political, and environmental factors to HIV vulnerability in specific contexts (Gupta et al. 2008; Hargreaves 2013). There are significant gaps in knowledge, however, about the development, implementation, and evaluation of structural interventions. First, while integration of food security interventions into HIV/AIDS prevention programs may be essential to curtail the HIV/AIDS pandemic and improve health and quality of life among those infected in resource-poor settings, (Weiser et al. 2011) the literature has offered little guidance to international policy makers, such as the World Food Programme (World Food Programme 2003). To our knowledge, there have been no published intervention studies examining the impact of economic status and food security on HIV outcomes in Malawi. Second, complex multilevel structural interventions are expensive. Typically, non-governmental organizations (NGOs) or government agencies implement them. The cost and complexity of study designs that would adequately evaluate real-world structural interventions do not align well with the typical NIH-funded randomized control trial (RCT) model; this presumably could explain the dearth of research. Third, major challenges remain in evaluating the impact of structural interventions. Few NGO interventions are evaluated rigorously to rule out alternative explanations of effects. Perhaps most importantly, few NGO program evaluations involve a control group. Further, most structural intervention assessments are limited to either structural variables on which they directly intervene (such as social norms that condone intimate-partner violence or microcredit program use rates) or key HIV health outcomes only (Gupta et al. 2008). These research gaps in the development, implementation, and evaluation of structural interventions limit their wider dissemination and scale-up in resource-poor countries, where services are desperately needed.

This manuscript describes the rationale, design, and methodology of a five-year study, called SAGE4Health, examining a structural, multilevel intervention carried out by a large NGO, CARE International, in rural Malawi. This longitudinal study represents one of the first attempts to understand the mechanisms and processes through which changes in food security and economic outcomes (i.e., income, household assets, livelihood options) can impact HIV vulnerability (i.e., HIV risk behaviors, malnutrition, HIV infections). It also represents one of the first NIH-funded studies based on an academic-economic development NGO partnership (Delisle et al. 2005; Weinhardt et al. 2009). This type of partnership leverages strengths of NGOs (i.e., their ability to respond quickly to crises and their capacity for large-scale, sustainable development work) and HIV/AIDS researchers’ rigorous study designs and evaluations. In addition to examining pathways linking distal ecosocial factors to HIV vulnerability, this study will provide important information for understanding the impact of multilevel structural interventions on HIV with the potential for sustainable long-term public health benefits. Finally, this collaboration provides a unique opportunity to conduct a detailed study of a multilevel intervention on a scale unlikely to be supported entirely by NIH research funding; in effect, we use the NIH and NGO program funding to enhance both contributions.

## Methods

### Overview of the study design, and participants

**Figure 1:**
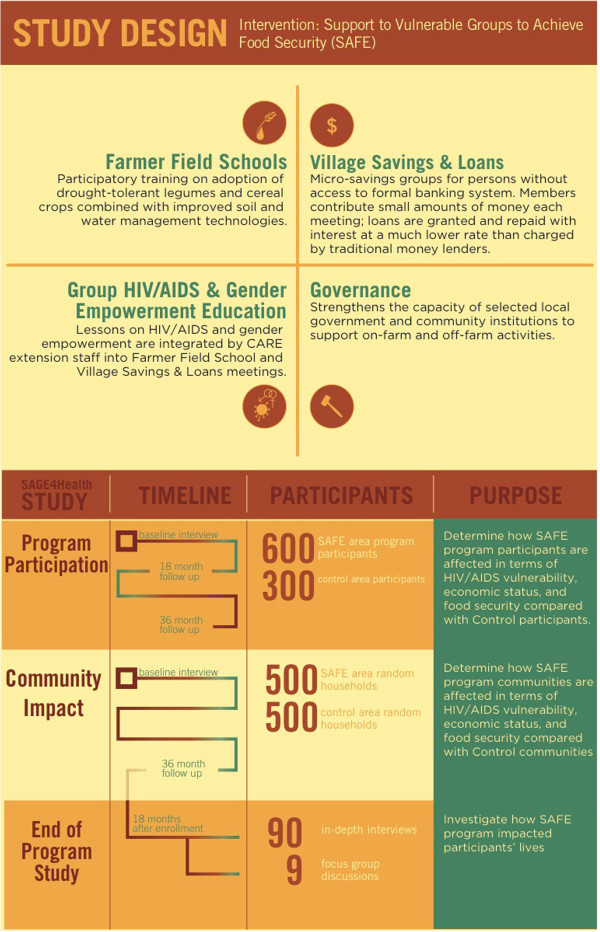
**SAGE4 Health Project study design and sampling procedure.**

**Figure 2:**
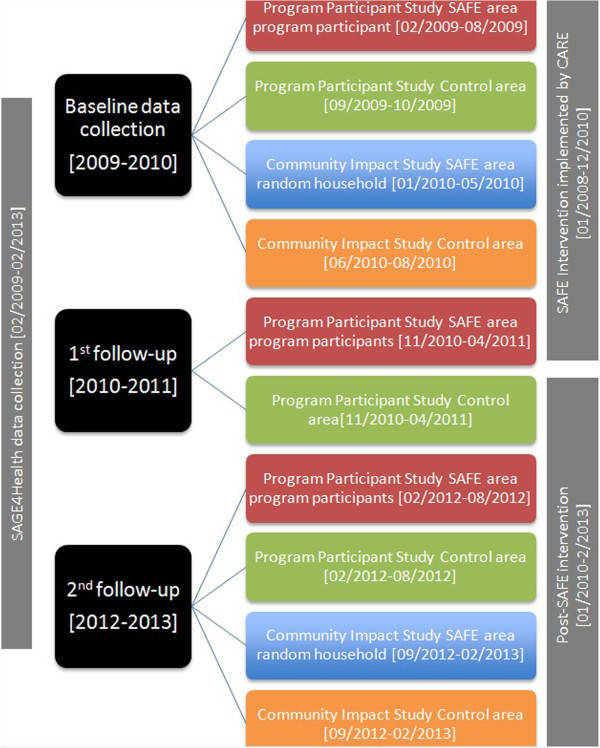
**SAGE4 Health Project study timeline.**

Our study, “SAGE4Health”, a five-year project, comprises *three interrelated* samples designed to systematically evaluate “Support to Able-Bodied Vulnerable Groups to Achieve Food Security (SAFE),” a community-based, structural, multilevel health and development intervention. Details about SAFE are described later. In February 2009, enrollment of households for the SAGE4Health study began in rural areas of the Lilongwe district of central Malawi. The overall goal of the study is to elucidate pathways between socioeconomic environment, food security, HIV risk behaviors and HIV-relevant outcomes. Figure 1 displays the SAGE4Health project study design and sampling procedure and Figure 2 shows the study timeline.

1. The first sample uses a longitudinal, quasi-experimental, nonequivalent-control group design (Shadish et al. 2001). The objective is to examine the magnitude and mechanisms of the impact of SAFE intervention on economic status, food security, HIV/AIDS vulnerability and other health-related outcomes at the SAFE program participants’ level. Participants (n = 598) from three Traditional Authorities (TAs) who received SAFE intervention (intervention group) are compared with 301 participants who live in three other matched TAs (control group, matched on demographics and distance from an urban center) not receiving SAFE. Quantitative data was collected in three waves: Baseline (during year 2009), 18-month follow up and 36-month follow up.
2. The second sample is derived from a random sample community survey with a cohort-sequential design (Prinzie and Onghena 2005; Whitley and Kite 2012). The strength of the cohort-sequential design is that it combines cross-sectional and longitudinal approaches by starting a new cohort each time an assessment is made. The objective is to check the possible threats (i.e., other external factors such as Malawi's national fertilizer and seed programs that were introduced in the same period as the intervention) to the internal validity of the intervention/evaluation by examining whether the intervention effects were the result of something plausible during the study period in the larger communities where the intervention was delivered. To achieve this objective, we randomly selected villages in SAFE TAs and continued interviewing all households in these villages until we had a sample of 500 households that were not direct participants in the SAFE program. We followed the same procedure for the control TAs, where the SAFE program had not been implemented. We conducted the same assessments as in sample one during the same time frame at baseline and 36-month follow-up.
3. The third sample consists of a series of in-depth qualitative interviews and focus groups conducted 18 months after enrollment ― near the end of SAFE program implementation. The objective is to understand SAFE participants’ experiences in the program, their perceptions of its impact and their perspectives on the phenomena under study ― food security, economic livelihood and HIV vulnerability.

The study was approved by the following Institutional Review Boards (IRBs): (a) Medical College of Wisconsin Human Research Review Committee and (b) National Health Sciences Research Committee, Malawi Ministry of Health. Written informed consent for participation in the study was obtained from all participants, and by parents/guardians for the children under 5 for the anthropometric measurements.

## Research team

The SAGE4Health research team consists of an interdisciplinary NGO-University partnership between CARE USA, CARE Malawi, the University of Wisconsin-Milwaukee (UWM) Joseph J. Zilber School of Public Health, the UWM College of Nursing’s Center for Global Health Equity, the Medical College of Wisconsin’s Center for AIDS Intervention Research, and the University of Malawi. The SAGE4Health study name reflects the key goals of the CARE’s SAFE Program: Savings, Agriculture, Governance and Empowerment. This partnership allowed the academic institutions and NGO to each contribute areas of expertise and learn from one another, rather than creating a costly situation whereby academia attempts to reinvent and implement an intervention in isolation. During the project, the academic collaborators provided the field staff, who had much experience in NGO-based field data collection but less experience in scientific research, with extensive in-person training in human subject research ethics, interviewing skills, data management, data analysis, scientific literature reviews, electronic data collection, participant tracking, oral presentation skills, and dissemination of results.

## Study setting

The study is being conducted in the rural areas of the Kasungu District of central Malawi. Among Malawian adults aged 15–49, approximately 11% live with HIV (National Statistical Office (NSO) and ICF Macro (2011)). Malawi’s population is young, rural, and poor National Statistical Office (NSO) and ICF Macro (2011) and 74% live below international poverty line of US$1.25 per day (UNICEF 2013). The Malawi economy is dominated by the agriculture sector, which employs 80% of the population, accounts for 42% of national GDP, supplies 81% of foreign exchange earnings and contributes significantly to national and household food security (Mataya 2003). Aside from agriculture, Malawi’s economy is also highly influenced by foreign aid. Based on the World Bank Africa Development Indicators 2011 Report, foreign aid accounted for 16.3% of Malawi’s GDP in 2009 (http://data.worldbank.org/sites/default/files/adi_2011-web.pdf). Given that Malawi’s economy receives substantial amounts of assistance, there is great interest from both donors and the Malawian government to understand the types of interventions that effectively create sustainable change in both the health and economic sectors.

### Overview of the CARE Malawi SAFE Intervention

We are evaluating the program “Support to Able-Bodied Vulnerable Groups to Achieve Food Security (SAFE),” which was developed and implemented from January 2008-December 2010 by CARE-Malawi, a country office of CARE International, a large NGO focused on ending poverty globally. The SAFE intervention was designed to address closely intertwined structural issues contributing to HIV susceptibility and poor health: food insecurity, poverty, gender inequity, and ineffective governance. The ultimate objective was to produce potentially sustainable improvements in the country’s socioeconomic fabric by facilitating those at greatest risk to move up a ‘ladder’ of interventions and services toward economic security and decreased health risks. SAFE was implemented in three TAs (Njombwa, Kaomba, and Mwase)—geographic subdivisions of Kasungu District, located in west-central Malawi. It was funded primarily by the European Commission and partially by the Austrian Development Cooperation.

The SAFE intervention had four main components (Table 1) addressing four major issues: (1) improving farming practices and sustainable agriculture through Farmer Field Schools, (2) increasing access to savings and investment through Village Savings and Loans Groups (VSL), (3) building capacity of local governance structures, and (4) integrating HIV education and gender empowerment into programs through training and education. Each component utilized feasibility studies, relationship building, and extensive community sensitization prior to implementation. Overall, more than 9,000 households were educated by Farmer Field Schools, 443 VSL groups formed and hundreds of village leaders trained regarding gender issues (Chima 2011). Details of each component, including objectives, activities, and outcomes are presented in Table 1.

**Table 1:** **SAFE Intervention four components**

Name of component	Objective	Output (participants, and activities)
Farmer Field School (FFS)	To improve crop production, and diversify income sources.	Through FFS, program participants practice improved farming activities, using an extension model, which promotes discovery-based learning through hands-on experimentation, critical thinking, and observation-based decision-making [24, 25].
		Actively demonstrating practices such as drought risk management, the improvement of seed input, and soil conservation practices, CARE extension agents planted crops in community “study fields.”
		Each FFS, consisting of approximately 25 farmers who share common farming experiences, meets regularly to follow the natural progression of the crop. These meetings included group dialogue, and reflection, as well as supplemental education sessions on topics such as HIV/AIDS, and gender empowerment training. Each improved farming activity was tested, validated, and adapted to local conditions.
Village Saving and Loan Groups (VSL)	To support food security through improving investment, and income earning opportunity at the household level.	VSLs are comprised of self-selected members who set the rules for the group.
		Trainers or “Village Agents” from CARE-trained, self-selected groups operate as a functional savings and loan group. Groups were to meet at regular intervals to save money by purchasing “shares” of savings.
Governance: capacity building of local governance structures, and community institutions	To support leaders at both the Traditional Authority and village levels to better affect project implementation and community development initiatives.	Leaders are trained to conduct problem analysis, planning, development, monitoring, and implementation linked to development of village action plans, using a community assessment-based scorecard process.
		Leaders utilized tools to map existing structures that support the community for improved food security. Then, to build upon these institutions, CARE trained the leaders in conducing district and community problem analysis.
Mainstreaming HIV/AIDS, and gender	To integrate issues of HIV and gender into all other program components.	Facilitators conduct a gender needs assessment for men and women in regard to VSL management, and crop production practices;
		Field staff is then trained to integrate gender empowerment and HIV information into in VSL management, farmer field schools, and within local institutions.

## SAGE4Health procedures

Below we delineate investigations using quantitative samples one and two, and the qualitative study, sample three. We discuss the assessment, power calculation (for quantitative studies only), and the data collection and analysis plan separately.

### Quantitative studies

#### Primary outcome assessment for quantitative investigation one and investigation two

**Figure 3:**
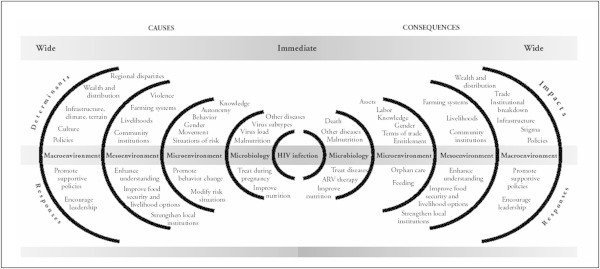
**Ecological framework of HIV determinants, impact, and responses.** (Loevinsohn and Gillespie, 2003).

Quantitative studies that involved samples one and two use the same survey instrument consisting of 23 modules (Table 2). We selected those measurement modules because they correspond to constructs of a social-ecological framework (Krieger 1994; Loevinsohn and Gillespie 2003) of HIV vulnerability that aligns with the multilevel nature of the SAFE intervention (Figure 3). The scales assess major structural variables, including economic status and food security, HIV vulnerability outcomes, such as self-reported HIV testing, infections, and individuals’ HIV/AIDS risk behavior and risk perceptions. In addition, gender empowerment and gender-based violence are measured. Also included is a verbal autopsy section to record and determine the cause of death of family members. Anthropometric measurements are taken to quantitatively assess the nutritional status of study participants and children under 5 in the household.

**Table 2:** **Questionnaire modules**

**1.**	Respondent characteristics
**2.**	Household sociodemographic characteristics
**3.**	Household economic status/livelihood strategies
**4.**	Housing and assets
**5.**	Use of assets for emergencies
**6.**	Income and expenses
**7.**	Household dietary diversity
**8.**	Household food security
**9.**	Household’s poverty perceptions
**10.**	Access to services
**11.**	Sustainable agriculture practices
**12.**	Personal health
**13.**	Illness occurrence and healthcare seeking
**14.**	Childbirth experiences
**15.**	Family planning
**16.**	Chronic illnesses
**17.**	Self-reported STD infections
**18.**	HIV/AIDS perception of risks, stigma and testing
**19.**	Male circumcision
**20.**	HIV risk activities
**21.**	Gender power
**22.**	Community cohesion
**23.**	Anthropometric measurements

Malawian field scientists administered the surveys face-to-face in Chichewa, the language spoken in the study area. Interview data were collected using Samsung Galaxy tablets utilizing the survey research platform Open Data Kit (Anokwa et al. 2009). The password-protected data were uploaded daily from the tablets to a desktop computer.

#### Major outcomes: Structural factors

##### Economic outcomes

Instruments measuring economic factors were adapted from the Malawi Diffusion and Ideational Change Project (MDICP) led by Watkins et al. (Anglewicz et al. 2009; Chin 2010) and previous CARE surveys. We focus selectively on important indicators of standard of living, consumption, production and income. Standard of living is measured by assets, income and consumption of basic commodities. Household production is measured by information on the quantities of major agricultural staples produced and the revenues of family enterprises in the past year. We also asked respondents about their relative wealth compared with other family members (specifically, siblings), and compared with other community members.

##### Food security, agricultural practices and nutrition outcomes

Food security is assessed with six questions from the preliminary CARE survey. These include questions about the months in the past year when the respondent’s household had enough food, projections for sufficient food crop production for the coming year and the household’s strategies when there was not enough food. Agriculture and natural resources are assessed with questions from CARE’s preliminary survey. For each of 23 sustainable agriculture practices (e.g., crop diversification, terracing, contour ridging, mulching), we ask respondents (1) if they know how to do it, (2) if they are applying it, and (3) if they are receiving benefit from it.

Nutrition is assessed with questions about the numbers of meals that household members ate in the past 24 hours, and 13 categories of foods consumed in the past 24 hours, with a yes/no response format. Anthropometric indicators including height, sitting height and weight are also collected for all respondents. Each measure is taken twice. If the difference is too large (e.g., > 1 cm for height), the measurement is repeated and an average of the two closest measures is used. BMI (Body Mass Index = weight/height^2^) and height-for-age (related to stunting) and weight-for-height (related to wasting) for children under the age of 5 are calculated, using the WHO/NCHS/CDC reference standards (de Onis et al. 2009, de Onis et al. 2006).

#### Major outcomes: HIV outcomes, HIV risk behavior and vulnerability variables

##### Subjective HIV testing, subjective HIV status and infection risk

Subjective HIV status and subjective HIV infection risks are elicited by having the respondents state the likelihood (from 0 = no risk to 10 = absolutely infected) that they (i) perceive themselves to be already infected, (ii) become infected in the next 5 years and (iii) ever become infected. These measures were developed for the MDICP (Anglewicz et al. 2009). HIV risk behavior (Weinhardt et al. 1999, 1998) measures (i.e., unprotected and protected sexual behaviors and sexually transmitted diseases) and a concise battery of measures derived from the Information-Motivation Behavior Skills Model (Fisher et al. 2002), as well as other relevant individual-level psychological factors, also were collected. HIV testing was assessed via self-report. Each participant was asked if they had ever been tested for HIV and, if so, what the result was.

### Power calculation

This study’s statistical power is determined for the primary HIV sexual risk outcome and the primary food security outcome. The primary study hypothesis was that the intervention group was associated with a greater increase in HIV testing and a greater increase in food security than the control group at the 18- and 36-month follow-ups. The analysis was based on longitudinal trajectory analysis comparing conditions. The sample size was 598 for the control group and 301 for the intervention group. Effect sizes (d) were given according to Cohen (1988). Based on the generalized estimating equations (GEE) (Liang and Zeger 1986) and the power calculation by Lin and Myers (2006), using a significance level of α = 0.05, the power to detect a moderate difference (effect size = 0.5) is over 90%.

### Data analysis for quantitative studies

Standard descriptive techniques are used to summarize and report study results. All data are inspected for skew and homogeneity of variance prior to data analysis. Data analysis of intervention outcomes is ongoing as of 2014. It is expected that distributions of some quantitative measures of sexual risk behavior data will be positively skewed, and transformations toward normality will be necessary. Non-parametric or generalized non-normality modeling techniques will be used for analyses if data are unable to be transformed to normal distributions. For hypothesis testing, the alpha level is set at 0.05.

Data analyses will utilize the full complexity (multilevel and repeated measures) of the data within and between individual households and between TAs. The strategy will use multiple analytical approaches: multivariate models, and generalized mixed linear models for longitudinal outcome data, coupled with structural equation modeling (SEM) of the pathways between primary outcomes and potential mediators suggested in an HIV ecosocial framework (Krieger 1994; Loevinsohn and Gillespie 2003).

This analysis plan is conceptualized as involving three phases: (1) repeated-measures analyses of HIV vulnerability, food security and economic outcomes assessed at baseline, and at 18- and 36-month intervals for participants in the SAFE participant- and non-SAFE comparison cohort; (2) analysis of HIV outcomes and vulnerability, food security and economic indicators from the random household community surveys in intervention and control TAs at baseline and at 36 months; (3) model-based mediation analysis for HIV vulnerability, food security and economic outcomes, based on the study cohort data. Furthermore, we will conduct gender moderator analysis. The purpose of gender moderator analysis is to examine whether the intervention has a different influence on men and women, and if so, why.

### Qualitative study: sample three

The objective of Sample 3 data collection and analysis is to understand SAFE participants’ perceptions of the intervention’s impact, and their perspectives on the major phenomena under study ― food security, economic livelihood and HIV vulnerability. This third sample consists of 90 SAFE participants (60 women and 30 men) from intervention group households (N = 600) who, at the end of the SAFE Program, participated in focus groups, as well as in-depth qualitative interviews about their intervention experiences. We used stratified purposive sampling (Ritchie et al. 2014) to mirror the composition of Sample 1, to represent equally the three TAs in which the intervention took place, and to capture diversity of exposure to all elements of the intervention. The nine focus groups and 90 individual interviews allowed us to reach data saturation and redundancy.

## Procedures

### Data collection

At the end of SAFE program implementation by CARE, audio-recorded focus groups and individual in-depth interviews were conducted in Chichewa face-to-face by the Malawian field scientists. The timing of the qualitative data collection with Sample 3 corresponded to the timing of the 18-month follow-up with Sample 1 (Table 3). The purposive sample of 20 women and 10 men from each of the three TAs (N = 90) participated in a total of nine sex-segregated focus groups and 90 interviews. Questions posed to the focus groups asked participants to reflect on how SAFE impacted them at a community level ― What changes had they observed in the village and larger community around them as a result of the intervention? They were also asked to discuss collectively the food security, economic well-being, and HIV risk circumstances they encountered in their communities before, during and at the close of the SAFE Program. In the individual interviews, participants were asked to describe the effects of SAFE at a personal level ― what changes had they observed in their own households as a result of the intervention? How did they personally assess the food security, economic well-being and HIV risk circumstances they dealt with every day?

**Table 3:** **Qualitative End-of-Program evaluation topics**

Focus group	In-depth interview
Activities of the SAFE Program?	SAFE activities you participated in?
What benefits? What problems for the village?	What benefits? What problems for you?
Perceived differences in the community after?	Perceived differences in the household after?
● Food security	● Food security
● Economic livelihood	● Economic livelihood
● Gender-based power	● Gender-based power
● HIV vulnerability	● HIV vulnerability

### Data analysis

These qualitative data were transcribed, translated into English, and verified and discussed with the field scientists, according to quality assurance guidelines. Qualitative data analysis strategies (Spradley 1979) are being applied to examine what changes in their communities, and in their households participants attribute to the SAFE intervention, and how they perceive the intervention to have worked. We are describing their viewpoints on food security, livelihood strategies, economic stability and HIV vulnerability; and identifying in their accounts how these structural dynamics operate in women’s and men’s lives.

We are using thematic analysis techniques (Braun and Clarke 2006; Mkandawire-Valhmu and Stevens 2010; Schensul et al. 1999) to code discrete units of meaning, chart the relationships among these units and describe the patterns of experience seen in the focus group data. Individual interview data are being analyzed narratively (Riessman 2008; Stevens and Galvao 2007; Stevens and Hildebrandt 2009), attending to the personal stories participants tell about their own experiences in relation to: a) obtaining food on a daily basis, b) securing income, and c) attempting to manage HIV risk. For each participant, we are coding story content and context in a dialectical process, examining reported events as well as participants’ interpretations of what happened to them. We will follow this within-case analysis with an across-case analysis in which we search for similarities and differences among participants in the stories they tell about how SAFE affected their daily lives. Combining within-case and across-case approaches produces more contextually grounded, transferable findings (Ayers et al. 2003). To further support the authenticity of findings and auditability of analytic processes, we are a) engaging in inter-rater reliability activities as we create and apply codes, b) writing memos about analytic decisions, and c) validating findings with the field scientists.

### Preliminary results

Preliminary analyses consist of baseline comparisons between the intervention and comparison groups in samples 1 and 2. Independent *t*-tests were used for continuous data and chi-squared tests were used for categorical data. Table 4 displays baseline comparisons. Participants in the intervention group (sample 1) did not differ substantially from those in the control group in terms of sociodemographic traits. However, there was evidence at baseline that participants in the intervention group were older (p = .040), had smaller sizes of households (p = .001) and the households were less dominated by males (p = .039). In terms of primary outcomes, intervention group and control group had significant differences in income sources (p = .025), self-report “ever tested for HIV” (p = .001), as well as practice/applying sustainable agriculture methods. Community random samples (sample 2) in the intervention and control areas were much the same in their sociodemographic profiles, except on household size and marital status. Differences in selected outcomes between community random samples in intervention and control areas were observed in terms of cash income source, self-report “ever tested for HIV” and sustainable agriculture methods practice/application.

**Table 4:** **Baseline characteristics of SAFE participant and community samples, by conditions (intervention vs. control)**

Characteristics	SAFE participant sample		P value	Random community sample		P value
	SAFE intervention	Control group		SAFE intervention area	Control area	
Demographics	N = 598	N = 301		N = 501	N = 501	
Female participants (%)	398 (66.6)	201 (66.8)	.947	334 (66.7)	327 (65.5)	.704
Mean age of respondent in years (range)	40.4 (18–84)	38.5 (19–86)	.040*	38.6 (17–84)	38.2 (3–98)	.658
Mean household size (range)	5.3 (1–11)	6.3 (2–14)	.001*	4.6 (1–13)	4.9 (1–12)	.021*
Male head of household	495 (82.8)	265 (88.0)	.039*	402 (80.24)	421 (84.2)	.101
Head of household literate	472 (78.9)	236 (78.4)	.856	375 (75.2)	363 (72.9)	.416
Marital status			.085			.021*
Currently married/living together	492 (82.3)	261 (86.7)		385 (77.0)	404 (80.8)	
Separated	21 (3.5)	9 (3.0)		18 (3.6)	8 (1.60)	
Divorced	19 (3.2)	11 (3.7)		48 (9.6)	27 (5.4)	
Widowed	54 (9.0)	20 (6.6)		44 (8.8)	54 (10.8)	
Never married	12 (2.00)	0 (0)		5 (1.0)	7 (1.4)	
Education (highest level of school)			.122			.353
Primary	447 (74.7)	225 (74.8)		366 (73.2)	383 (76.9)	
Secondary	81 (13.5)	28 (9.3)		62 (12.4)	49 (9.8)	
University	0 (0)	0 (0)		0 (0)	1 (0.2)	
Other	2 (0.3)	1 (0.3)		0	0	
Never went to school	68 (11.4)	47 (15.6)		72 (14.4)	65 (13.1)	
Have multiple spouses (%)	68 (21.8)	41 (24.3)	.537	42 (16.7)	57 (22.5)	.097
N of missing	286	132		249	248	
**Most important source of livelihood**			.688			.140
Crop farming	541 (90.5)	277 (92.0)		443 (88.4)	440 (87.8)	
Casual labor/ganyu	16 (2.7)	11 (3.7)		36 (7.2)	30 (6.0)	
**Most important cash income source**			.025*			.001*
Crop farming	372 (62.2)	203 (67.4)		285 (57.0)	356 (71.1)	
Casual labor/gangyu	72 (12.0)	33 (11.0)		105 (21.0)	79 (15.8)	
Trading/selling	17 (5.6)	64 (10.7)		31 (6.20)	37 (7.39)	
**Food insecurity (≥one month with insufficient food, past 12 months)**	426 (71.2)	218 (72.7)	.654	378 (75.4)	384 (76.8)	.616
**Ever tested for HIV**	316 (52.8)	195 (64.8)	.001*	323 (64.7)	291 (58.6)	.045*
**Self-report HIV-positive test**	17 (5.6)	11 (5.8)	.929	21 (6.6)	10 (3.5)	.084
**Practice/applying sustainable agriculture methods**						
Compost manure	289 (48.3)	131 (43.5)	.001*	160 (32.0)	104 (20.8)	.001*
Kraal manure	348 (58.2)	161 (53.5)	.001*	221 (44.2)	220 (43.9)	.745
Crop residue/vegetation incorporation	303 (50.7)	133 (44.2)	.013*	183 (36.5)	166 (33.2)	.067
Terracing	2 (0.3)	4 (1.3)	.171	2 (0.4)	13 (2.6)	.024*
Marker Ridges	185 (30.9)	18 (6.0)	.001*	81 (16.2)	72 (14.4)	.456
Box ridges	272 (45.5)	136 (45.2)	.001*	92 (18.4)	87 (17.4)	.686
Dams	7 (1.2)	1 (0.33)	.001*	3 (0.6)	2 (0.4)	.548
Crop diversification	561 (93.8)	273 (90.7)	.063	410 (82.0)	405 (80.8)	.630
Seed multiplication	274 (45.8)	87 (28.9)	.001*	155 (31.1)	132 (26.3)	.171
Drought tolerant and early maturing crops	390 (65.2)	197 (65.4)	.045*	247 (49.4)	300 (59.9)	.001*
Legumes with cereals	435(72.7)	253 (84.1)	.001*	278 (55.7)	375 (74.9)	.001*
Fruit production	296 (49.5)	150 (49.8)	.277	218 (43.5)	263 (52.6)	.004*
Vegetable production	330 (55.2)	207 (68.8)	.001*	171 (34.1)	337 (67.5)	.001*
Home gardens-indigenous herbs and vegetables	63 (10.5)	25 (8.3)	.155	27 (5.4)	83 (16.6)	.001*
Cover cropping	3 (0.5)	0 (0)	.219	6 (1.2)	13 (2.6)	.082
Crop rotation	546 (91.3)	275 (91.4)	.351	375 (74.9)	418 (83.4)	.002*
Irrigation farming	252 (42.1)	186 (61.8)	.001*	158 (31.7)	308 (61.6)	.001*
Improved grain storage	340 (56.9)	195 (64.8)	.039*	300 (59.9)	336 (67.1)	.039*

* = *p*<.05.

## Discussion

In linking academic research to a “real world,” NGO-led structural intervention, this study design may yield many benefits. First, the cost sharing of the implementation and the research between the NGO and the academic institution allowed for a fully-funded project that may not have been possible had only one funding stream been available. In an environment of constricting research funding, it is prudent to develop such mutually beneficial partnerships. Furthermore, the NGO-academia relationship extended beyond a grant application into capacity building. The NGO field staff developed expertise in research methods, including study protocols, human research ethics trainings, institutional review board (IRB) procedures, sampling methods, qualitative interview skills, data management techniques and computer-assisted quantitative interview skills; the academic staff gained insight into the logistics of on-the-ground program implementation, including international IRB processes, multi-agency grants management, logistics planning in challenging environments, NGO operations and interdisciplinary communication.

Due to the rapid pace with which NGOs undertake programs, compared to the relatively slow pace of federally funded academic research and result dissemination, this partnership has the potential to lead to rapid use of results. Given the program ownership by the NGO, the organization and its funders have a vested interest in understanding the effectiveness, or lack thereof, of its interventions. As the NGO and academic institution work in partnership, results are fed back to the implementers on a more real-time basis. The NGO, thus, has the opportunity to adjust future programming based on this study’s findings.

Limitations of this study include the use of self-reported outcomes and the quasi-experimental design. There is no random assignment to condition at the individual or community level. As such, the intervention and control groups may systematically differ in factors other than intervention exposure. As shown in Table 4, however, despite the nonequivalent-control group design, the intervention and control groups were very similar at baseline on key outcome variables. Another limitation of our design is that we cannot measure the separate effects of each intervention component. However, we feel the benefits of understanding the effects of the program as a whole, as designed, outweigh the benefit of a randomized treatment dismantling design. Bonell et al. (2006), Victora et al. (2004) and others have proposed designs such as ours to evaluate complex interventions in field studies.

There are many perspectives on whether structural interventions and public health should be evaluated using the RCT. Victora et al. argue that blind acceptance of RCT designs may not provide the entire picture of an intervention’s effects (Victora et al. 2004). They note, “Current trends toward acceptance of RCTs as the gold standard source of evidence may limit the knowledge base needed to make sound decisions about public health priorities, and policies” (Victora et al. 2004). External validity and generalizability are key to evaluations of the type presented here, as the partner NGO has the power to utilize findings to make programming changes, as well as replicate the intervention to other areas. RCTs are not the ideal study design when equipoise does not exist, where the intervention is already underway, and where tailoring of the intervention is not possible — for example, it must be consistent across an entire country as in some educational programs (Bonell et al. 2011). Though it would have been possible to construct an RCT of the SAFE intervention, it would not have fit into the established NGO mode of operation.

In addition to examining pathways linking environmental factors to HIV vulnerability, this research will yield important information for understanding the impact of multilevel environmental/structural interventions on HIV with the potential for sustainable long-term public health benefits. Finally, this collaboration represents a unique opportunity to conduct a rigorous effectiveness study of a multilevel intervention being implemented on a scale that is unlikely to be supported entirely by NIH research funding; in effect, we are leveraging the NIH and NGO program funding to enhance the contributions of both.

## Abbreviations

AIDS: Acquired immune deficiency syndrome
ARV: Anti-retroviral
FFS: Farmer Field Schools
GDP: Gross domestic product
HIV: Human Immunodeficiency Virus
IRB: Institutional Review Board
NGO: Non-governmental organization
NIH: National Institutes of Health
RCT: Randomized controlled trials
SAFE: Support to able bodied vulnerable groups to achieve food security
SAGE4Health: Savings, Agriculture, Governance & Empowerment for Health Study
TA: Traditional Authorities
UWM: University of Wisconsin Milwaukee
VSL: Village Savings and Loans.
